# Changes in Proteasome Structure and Function Caused by HAMLET in Tumor Cells

**DOI:** 10.1371/journal.pone.0005229

**Published:** 2009-04-14

**Authors:** Lotta Gustafsson, Sonja Aits, Patrik Önnerfjord, Maria Trulsson, Petter Storm, Catharina Svanborg

**Affiliations:** 1 Institute of Laboratory Medicine, Department of Microbiology, Immunology and Glycobiology, Lund University, Lund, Sweden; 2 Department of Experimental Medical Science, Lund University, Lund, Sweden; 3 Singapore Immunology Network (SIgN), Biomedical Sciences Institutes, Agency for Science, Technology, and Research (A*STAR), IMMUNOS, BIOPOLIS, Singapore, Singapore; Health Canada, Canada

## Abstract

**Background:**

Proteasomes control the level of endogenous unfolded proteins by degrading them in the proteolytic core. Insufficient degradation due to altered protein structure or proteasome inhibition may trigger cell death. This study examined the proteasome response to HAMLET, a partially unfolded protein-lipid complex, which is internalized by tumor cells and triggers cell death.

**Methodology/Principal Findings:**

HAMLET bound directly to isolated 20S proteasomes *in vitro* and in tumor cells significant co-localization of HAMLET and 20S proteasomes was detected by confocal microscopy. This interaction was confirmed by co-immunoprecipitation from extracts of HAMLET-treated tumor cells. HAMLET resisted *in vitro* degradation by proteasomal enzymes and degradation by intact 20S proteasomes was slow compared to fatty acid-free, partially unfolded α-lactalbumin. After a brief activation, HAMLET inhibited proteasome activity *in vitro* and in parallel a change in proteasome structure occurred, with modifications of catalytic (β1 and β5) and structural subunits (α2, α3, α6 and β3). Proteasome inhibition was confirmed in extracts from HAMLET-treated cells and there were indications of proteasome fragmentation in HAMLET-treated cells.

**Conclusions/Significance:**

The results suggest that internalized HAMLET is targeted to 20S proteasomes, that the complex resists degradation, inhibits proteasome activity and perturbs proteasome structure. We speculate that perturbations of proteasome structure might contribute to the cytotoxic effects of unfolded protein complexes that invade host cells.

## Introduction

Cellular quality control systems survey protein synthesis to ensure that erratic or unfolded products are degraded. The proteasomes are crucial for extra-lysosomal protein degradation taking place in the barrel-shaped 20S proteasome core [1]–[4]. The proteasome response to endogenous unfolded proteins has been extensively studied and proteasome inhibition and the failure to eliminate erratic proteins is a well-known cause of amyloid formation and disease [5], [6]. In addition, the disease-causing prion protein oligomers were recently shown to inhibit the 26S proteasome [7] but except for this study, little is known about unfolded extracellular proteins that enter target cells, and about the involvement of proteasomes in the cytotoxic response to such proteins.

HAMLET (human α-lactalbumin made lethal to tumor cells) is a protein-lipid complex that kills tumor cells [8], [9] and shows promising activity *in vivo* [10], [11]. Intra-cranial infusion of HAMLET prolonged survival in rats carrying human glioblastoma xenografts [11] and intra-vesical HAMLET inoculation in bladder cancer patients caused rapid shedding of tumor cells and a reduction in tumor size [10]. Topical HAMLET application removed skin papillomas in a placebo-controlled clinical study [12]. The mechanism(s) of tumor cell death are not fully understood, however.

Mature, folded α-lactalbumin acts as a coenzyme in lactose synthesis [13]–[15] but partially unfolded α-lactalbumin forms HAMLET by incorporating oleic acid [9]. The fatty acid cofactor is essential for the tumoricidal activity since it helps maintain the partially unfolded state in cellular environments [9], [16]. The tendency of α-lactalbumin to form stable folding intermediates is well-known [17]–[19] but until the discovery of HAMLET, changes in tertiary structure had not been proposed to alter the function of the protein. Structural studies have suggested that the conformational change of α-lactalbumin to a partially unfolded state is necessary for the tumoricidal effect of HAMLET [16].

While *in vitro* studies have suggested that 20S proteasomes degrade partially unfolded α-lactalbumin [20], cellular distribution studies have shown that HAMLET accumulates in tumor cells, suggesting that the mechanisms used by these cells to digest unfolded α-lactalbumin are inefficient against HAMLET. We hypothesized that internalized HAMLET might be identified as partially unfolded α-lactalbumin and targeted to the proteasomes for degradation [21] but that bound oleic acid might reduce the sensitivity of partially unfolded α-lactalbumin to proteasome degradation, making the HAMLET complex potentially toxic for the proteasome.

This study examined if HAMLET interacts with and modifies tumor cell proteasomes. The results suggest that HAMLET interacts directly with 20S proteasomes, modifies their structure and inhibits their activity.

## Results

**Figure 1:**
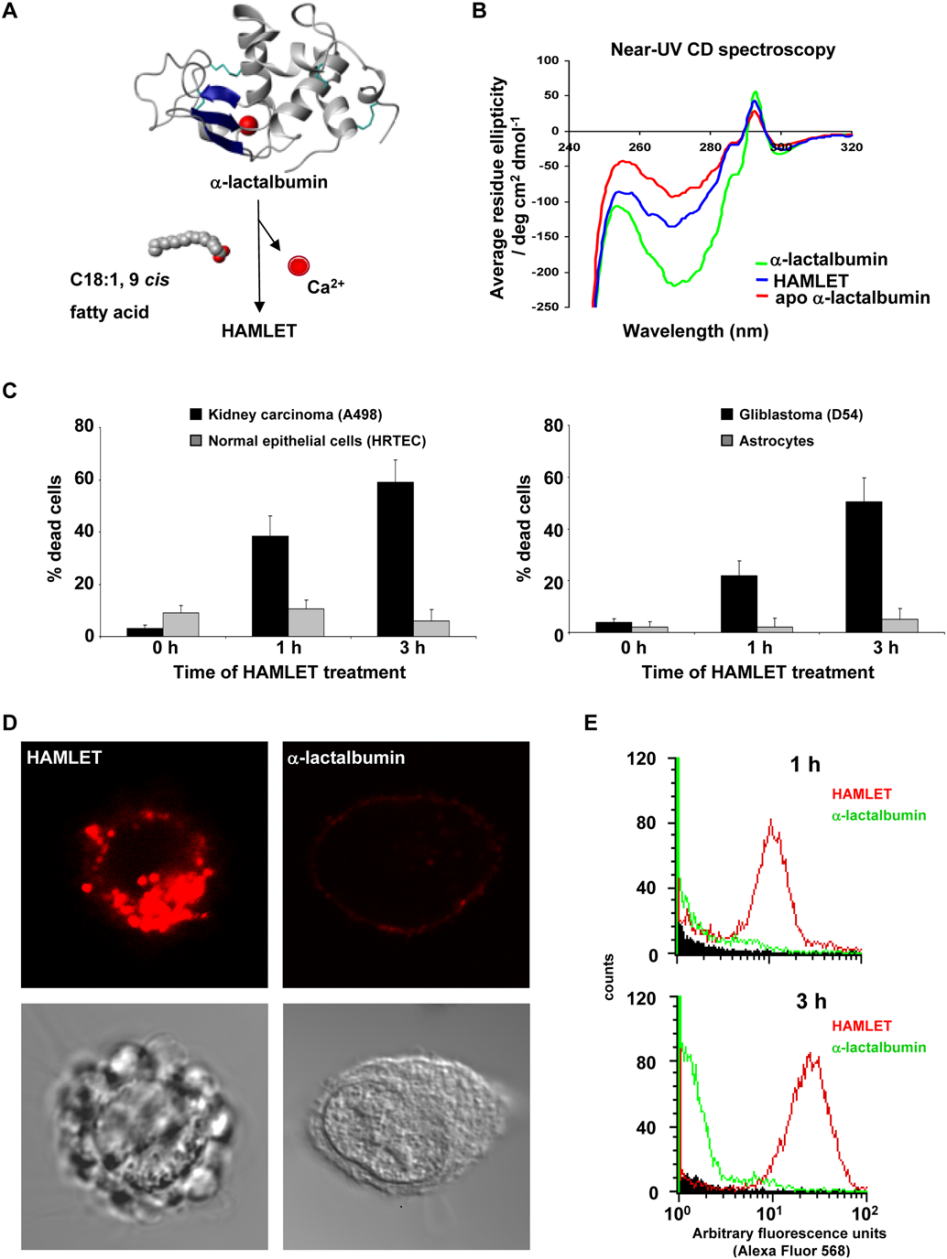
HAMLET is a complex of partially unfolded α-lactalbumin and oleic acid. (A) HAMLET is formed when native α-lactalbumin releases the strongly bound Ca^2+^ and exposes a fatty-acid binding site, which permits oleic acid (C18:1, 9 *cis*) to interact and stabilize the partially unfolded state. The figure was generated using the crystal structure of human α-lactalbumin [39] and MOLMOL 2K.2 [40]. (B) Near-UV CD spectra were recorded in sodium phosphate buffer without EDTA. Native α-lactalbumin showed the expected minimum at 270 nm arising from the tyrosine and a maximum at 293 nm arising from the tryptophan residues. In HAMLET, α-lactalbumin showed a loss of signal at 270 nm and at 293 nm, typical of partial unfolding [9], [16]. Apo α-lactalbumin was obtained by EDTA treatment and showed a similar loss of signal. (C) Effect of HAMLET on tumor cells and healthy cells. Glioblastoma cells and kidney carcinoma cells treated with HAMLET (34 µM, 1 and 3 hours) died while normal astrocytes and renal epithelial cells (HRTEC) remained viable. (D) Difference in uptake of HAMLET and α-lactalbumin by tumor cells. Lung carcinoma cells (A549) were exposed to 34 µM of Alexa Fluor 568-labeled HAMLET or α-lactalbumin (both red). (E) Quantification by flow cytometry of HAMLET or α-lactalbumin uptake by A549 cells (N = 10,000) after 1 and 3 hours of incubation.

α-Lactalbumin is secreted by the mammary gland as a mature, folded protein but can unfold by releasing Ca^2+^ [22]–[24]. Extracellular unfolding can occur for example at low pH [25] in environments like the stomach. To produce the HAMLET complex in the laboratory, native α-lactalbumin is partially unfolded with EDTA to the apo state and then converted to HAMLET on an oleic acid-preconditioned ion exchange matrix (Figure 1A) [9]. The fatty acid stabilizes the protein in the partially unfolded state, as shown by near-UV CD spectroscopy (Figure 1B) [9].

The tumoricidal activity of HAMLET is shown in Figure 1C. Human glioma cells and kidney carcinoma cells died rapidly in response to HAMLET (34 µM) but normal differentiated cells from the same tissues were resistant to the lethal effect (Figure 1C).

The internalization of HAMLET by tumor cells is visualized in Figure 1D, using Alexa Fluor 568-labeled HAMLET (34 µM, 1 hour) in confocal microscopy. Tumor cells (A549) showed strong intracellular staining after 1 hour and the cells changed morphology and died. Native α-lactalbumin bound less efficiently than HAMLET to the tumor cell surface, only small amounts were internalized and the cells remained viable (Figure 1D). The difference in uptake between HAMLET and α-lactalbumin was quantified by flow cytometry. In tumor cells, there was a ∼50 fold difference in uptake after 1 hour between HAMLET and α-lactalbumin (Figure 1E). There was also a marked difference in HAMLET uptake measured by confocal microscopy between tumor cells and normal cells of the same tissue origin. Rapid internalization of HAMLET was detected in glioblastoma cells (D54) and kidney carcinoma cells (A498) but not in normal kidney epithelial cells (HRTEC). Normal astrocytes internalized small amounts of HAMLET (data not shown).

### HAMLET interacts with 20S proteasomes

**Figure 2:**
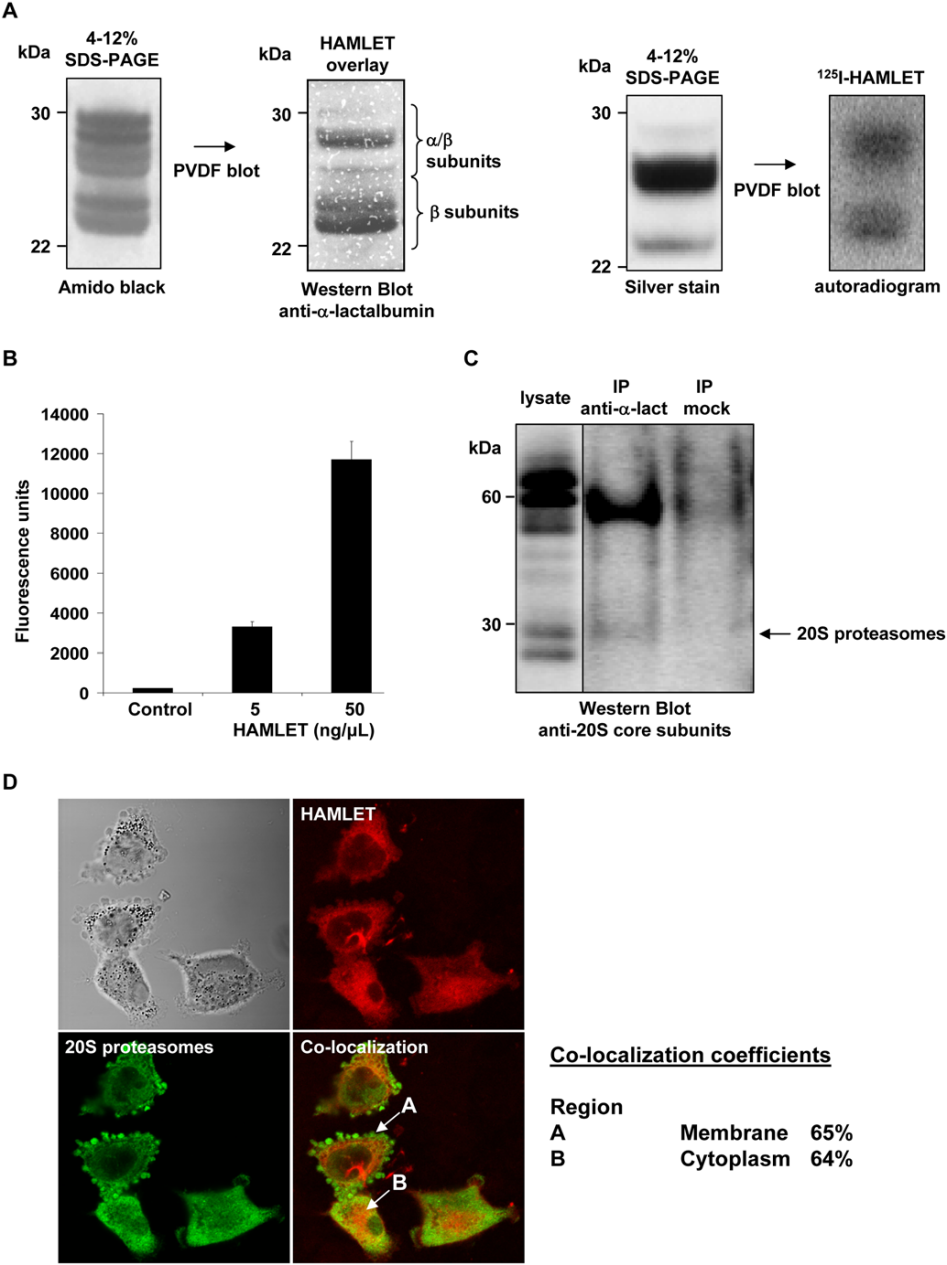
Interaction of HAMLET with proteasomes *in vitro* and in tumor cells. (A) Binding of HAMLET to 20S proteasome subunits. 20S proteasomes were denatured, separated by SDS-PAGE and stained with Amido black or silver staining or blotted onto PVDF membranes. Left panels: Membranes were overlaid with 0.14 mM HAMLET and binding was detected by immunoblot using anti-α-lactalbumin antibodies. Right panels: Membranes were overlayed with ^125^I-HAMLET and binding was detected by autoradiography. (B) Binding of HAMLET to 20S proteasome β5 subunit on a protein array. Protein arrays were probed with Alexa Fluor 568-labeled HAMLET and fluorescence intensity was measured. HAMLET bound to the 20S proteasome β5 subunit in a concentration-dependent manner. (C) Co-immunoprecipitation of lysates from HAMLET-treated A549 cells (34 µM, 1 hour) with anti-α-lactalbumin antibodies (IP anti-α-lact) or no antibodies (IP mock). Lysates and precipitates were examined by Western Blot using antibodies against the 20S proteasome core subunits. A very weak band was detected at ∼30 kDa. (D) Confocal images showing co-localization of Alexa Fluor 568-labeled HAMLET (34 µM, 1 hour) and 20S proteasomes (antibody against 20S proteasome core subunits) in the cell periphery and cytoplasm of A549 cells.

To examine the *in vitro* interaction between HAMLET and 20S proteasomes, intact 20S proteasomes (6 µg) were denatured and the subunits were separated by SDS-PAGE and blotted onto PVDF membranes. The membranes were overlaid with HAMLET (0.14 mM) and stained with polyclonal anti-α-lactalbumin antibodies. HAMLET was shown to bind to proteasome subunits ranging in molecular weight from about 22–30 kDa (Figure 2A, left panel). Primary and secondary antibody controls were negative. The *in vitro* binding was confirmed by overlay of the blotted membranes with radio-labeled HAMLET (Figure 2A, right panel). These results suggest that HAMLET and 20S proteasome subunits can interact.

Further evidence of the binding of HAMLET to proteasome subunits was obtained using a protein array. Arrays probed with HAMLET were compared to negative controls without HAMLET. One of the top-scoring hits, with a Z-score of 6.54, was proteasome subunit β5 (Figure 2B). This hit showed very low variation between assays (coefficient of variation 8%) and replicate spots (coefficient of variation 1%). Proteasomal subunits α1, 3 and 6 and β3 also showed increasing signal intensity with rising HAMLET concentrations although they did not reach the statistical threshold used to define a positive hit.

To examine if HAMLET interacts with 20S proteasomes in tumor cells co-immunoprecipitation was used. A549 cells were treated with HAMLET (34 µM, 1 hour), lysed and HAMLET and associated proteins were precipitated from the lysates with anti-α-lactalbumin antibodies. Lysates and precipitates were examined for the presence for 20S proteasomes by Western blots with antibodies against the 20S core subunits (α5, α7, β1, β5, β5i and β7). A very weak band of ∼30 kDa was recognized in the precipitate from HAMLET-treated cells (Figure 2C). In addition the antibodies against the 20S core subunits recognized bands of ∼60 kDa in the lysate and precipitate from HAMLET-treated cells. These bands could not be identified but might represent complexes of HAMLET and 20S proteasomes.

The interaction of HAMLET with 20S proteasomes was visualized by confocal microscopy (Figure 2D). A549 cells on microscope slides were treated with Alexa Fluor 568-labeled HAMLET (34 µM, 1 hour), fixed and stained with antibodies against the 20S core subunits. Co-localization of HAMLET and 20S proteasomes was detected in the cell periphery (65% co-localization) and throughout the cytoplasm of adherent cells (64% co-localization, Figure 2D).

### Resistance of HAMLET to degradation by proteasomal enzymes

**Figure 3:**
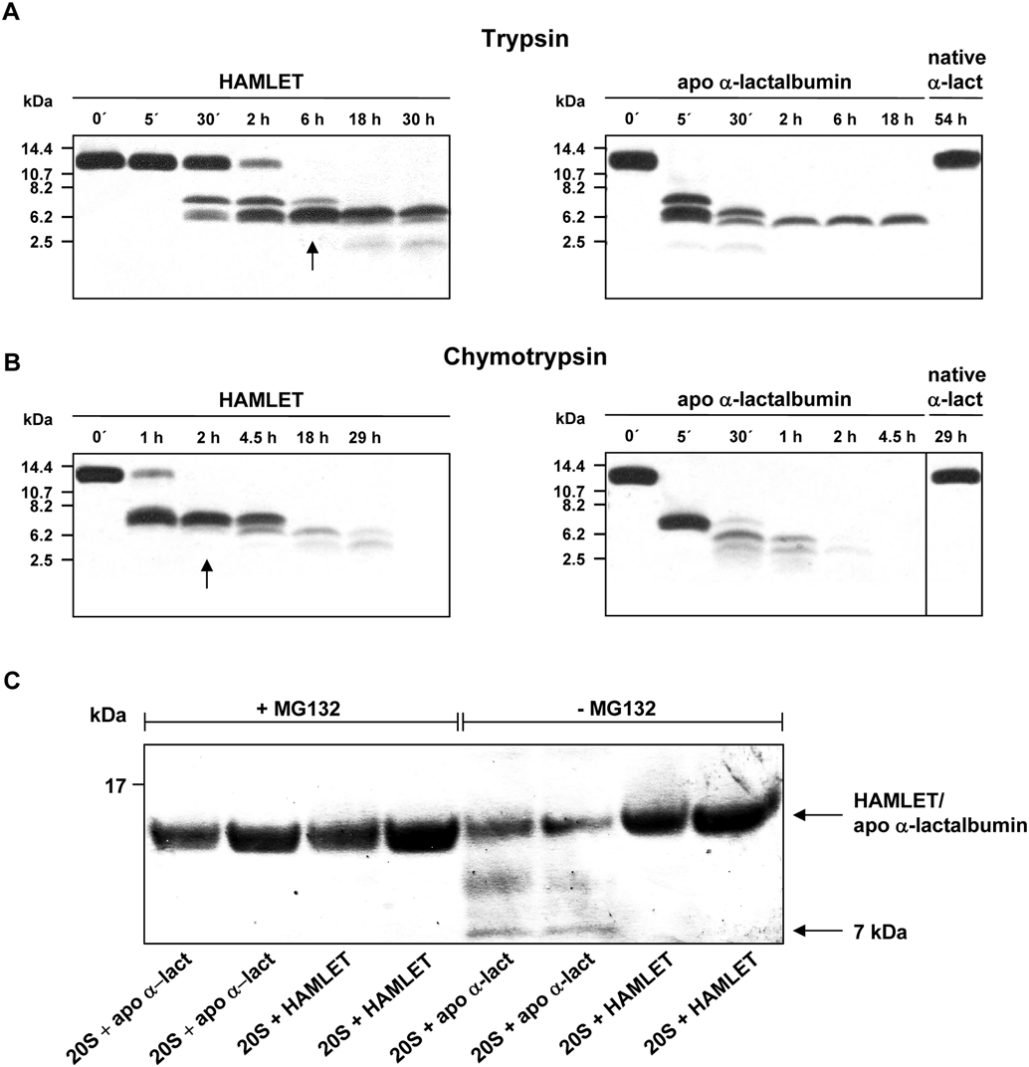
Resistance of HAMLET to degradation by proteasomal enzymes. (A, B) HAMLET, partially unfolded α-lactalbumin (apo α-lactalbumin) or native α-lactalbumin was exposed to trypsin (E∶S 1∶50, by weight) (A) or chymotrypsin (E∶S 1∶100, by weight) (B) *in vitro* and fragments were detected by SDS-PAGE. Native α-lactalbumin was in 200 µM calcium acetate whereas partially unfolded α-lactalbumin was in 200 µM EDTA. Arrows indicate time points when all HAMLET was degraded. (C) HAMLET or partially unfolded α-lactalbumin (apo α-lact) (both 7 µM) were mixed with 6 µg of human erythrocyte 20S proteasomes and incubated for 2 hours at 37°C. The proteins were separated by SDS-PAGE and visualized by silver staining. MG132 was used to inhibit proteasome activity. The arrows indicate the molecular sizes of full length apo α-lactalbumin/HAMLET (14 kDa) and a smaller fragment (∼7 kDa).

The sensitivity to proteolytic degradation was compared between HAMLET, native α-lactalbumin and partially unfolded, fatty acid-free α-lactalbumin (EDTA-treated). The proteins were exposed to trypsin or chymotrypsin, samples were obtained after 5 minutes and at various time points until 54 hours and fragmentation was examined by SDS-PAGE. Partially unfolded α-lactalbumin was completely degraded after 5 minutes exposure to trypsin (Figure 3A) or chymotrypsin (Figure 3B). HAMLET started to degrade after 30 minutes exposure to trypsin and after 1 hour exposure to chymotrypsin but complete degradation required at least 2 hours for chymotrypsin and 6 hours for trypsin (see arrows in Figure 3A, B). The native protein was resistant to degradation and remained intact after 54 and 29 hours exposure to trypsin and chymotrypsin, respectively.

The sensitivity of HAMLET to degradation was further examined by *in vitro* incubation of HAMLET or partially unfolded EDTA-treated α-lactalbumin (7 µM) with active 20S proteasomes (6 µg) for 2 hours, followed by SDS-PAGE and silver staining. Two different batches of HAMLET and α-lactalbumin were used. Both of the two EDTA-treated, partially unfolded α-lactalbumin samples were degraded after 2 hours and degradation was blocked by the chymotrypsin inhibitor MG132. In contrast, no degradation of the HAMLET samples was detected (Figure 3C). The results suggested that HAMLET is more resistant to proteolytic degradation than partially unfolded α-lactalbumin protein without oleic acid.

### Effects of HAMLET on proteasome activity

**Figure 4:**
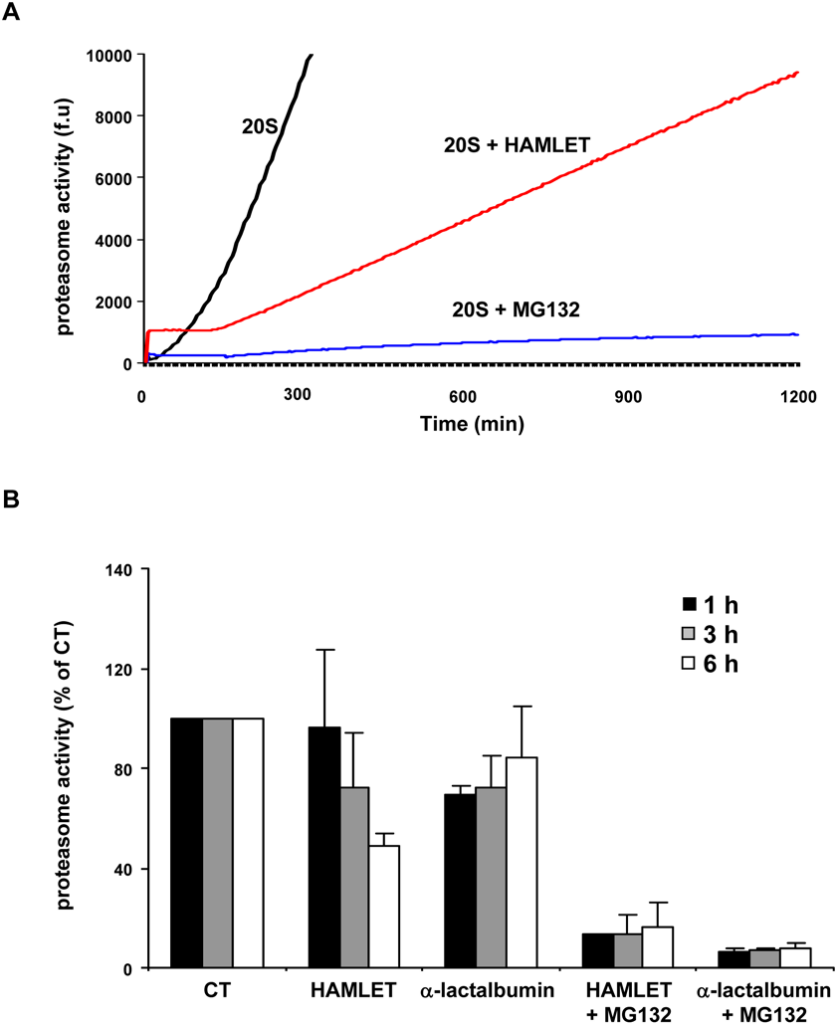
HAMLET inhibits proteasome activity. (A) Proteasome activity was monitored *in vitro* as chymotrypsin-dependent suc-LLVY-AMC cleavage (50 µM) over time and MG132 was used as proteasome inhibitor. Except for the first 10 minutes HAMLET reduced proteasome activity compared to the control. (B) Inhibition of proteasome activity in A549 cells. Cytoplasmic extracts were prepared from cells treated with HAMLET or native α-lactalbumin (34 µM) after 1, 3 and 6 hours of incubation and enzymatic activity was quantified by suc-LLVY-AMC cleavage (50 µM). MG132 was used to inhibit proteasome function.

To examine if HAMLET influenced proteasome function, we quantified the proteasome activity *in vitro*, using isolated 20S proteasomes (200 ng) and chromogenic substrates (LLVY-AMC, 50 µM). The proteasomes were enzymatically active as shown by substrate cleavage in the control and this activity was inhibited by MG132 (50 µM, Figure 4A). HAMLET caused a low, initial burst of proteasome activity lasting about 10 minutes but subsequently HAMLET acted as a partial proteasome inhibitor compared to the control (Figure 4A). The results indicated that HAMLET impaired the catalytic activity of the proteasome core. This effect was confirmed in extracts from HAMLET-treated A549 carcinoma cells (34 µM, 1, 3 and 6 hours). A significant reduction in proteasome activity was detected after 3 and 6 hours of HAMLET treatment (Figure 4B, p<0.01, 6 hours).

### Structural modifications of 20S proteasomes

**Figure 5:**
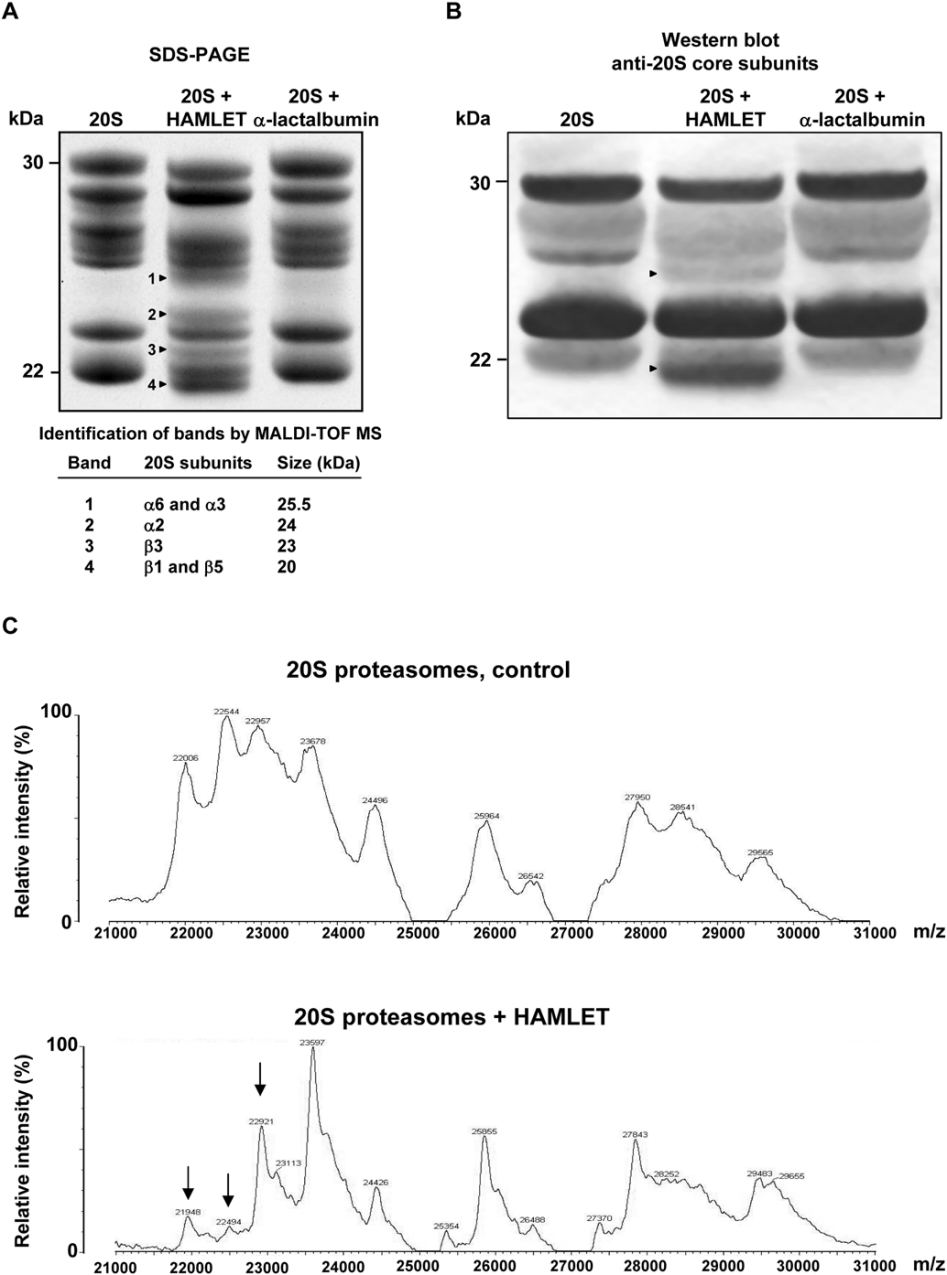
Structural changes in 20S proteasomes after HAMLET treatment *in vitro*. (A, B) *In vitro* fragmentation of human 20S proteasomes after 20 minutes incubation with HAMLET (7 µM), compared to the α-lactalbumin control. (A) The proteins were separated by SDS-PAGE and gels were stained with Amido black. Changes in 20S proteasome subunits are indicated by arrows. The subunits identified by mass spectrometry are shown in the table. (B) Western Blot of samples from *in vitro* mixing experiment with 20S proteasomes and HAMLET or α-lactalbumin using antibodies against the 20S proteasome core subunits. (C) MALDI-TOF mass spectrometry of intact 20S proteasomes (top panel) and major changes after HAMLET treatment (bottom panel), indicated by arrows.

To examine if HAMLET might alter proteasome structure, HAMLET (7 µM) was mixed with intact 20S proteasomes (6 µg) *in vitro* for 20 minutes and the samples were run on SDS-PAGE (12% gel) under denaturing conditions. Structural modifications of the proteasomes were detected by Amido black staining and altered bands were trypsinized and identified by mass spectrometry (Figure 5A). Four new bands were identified (see arrows, Figure 5A). Band 1 contained the structural α3 subunit and the α6 subunit that contains the nuclear localization sequence KKXK. Band 2 contained the structural α2 subunit and band 3 the catalytic β3 subunit. Band 4 contained a mixture of the catalytic subunits β1 with peptidyl glutamyl hydrolyzing activity and β5 with chymotrypsin-like activity. The observed protein matches were significant (p<0.001). In order to investigate if autocatalytic chymotrypsin activity was responsible for the proteasome fragmentation, non-tryptic peptide masses were searched against semi-chymotrypsin cleavages using the FindPept program. One peptide from the α2 subunit was matched (data not shown), suggesting that the changes in proteasome structure might arise from both chymotrypsin-dependent and other potentially autocatalytic activities.

Proteasome fragmentation in response to HAMLET was further examined by Western blot (4–12% gel) (Figure 5B). Antibodies against the 20S proteasome core subunits recognized a new proteasome band of about 26 kDa and one of about 20 kDa (see arrows, Figure 5B).

The change in 20S proteasome structure was confirmed by mass spectrometry analysis of *in vitro* mixtures of HAMLET (7 µM) and proteasomes (6 µg) (Figure 5C). The major difference between the spectra is the decreased relative peak height for masses below 23.6 kDa. Minor other differences were observed. However, the mass accuracy and resolution did not allow identification of individual subunits based on mass alone.

### Changes in proteasome structure in tumor cells

**Figure 6:**
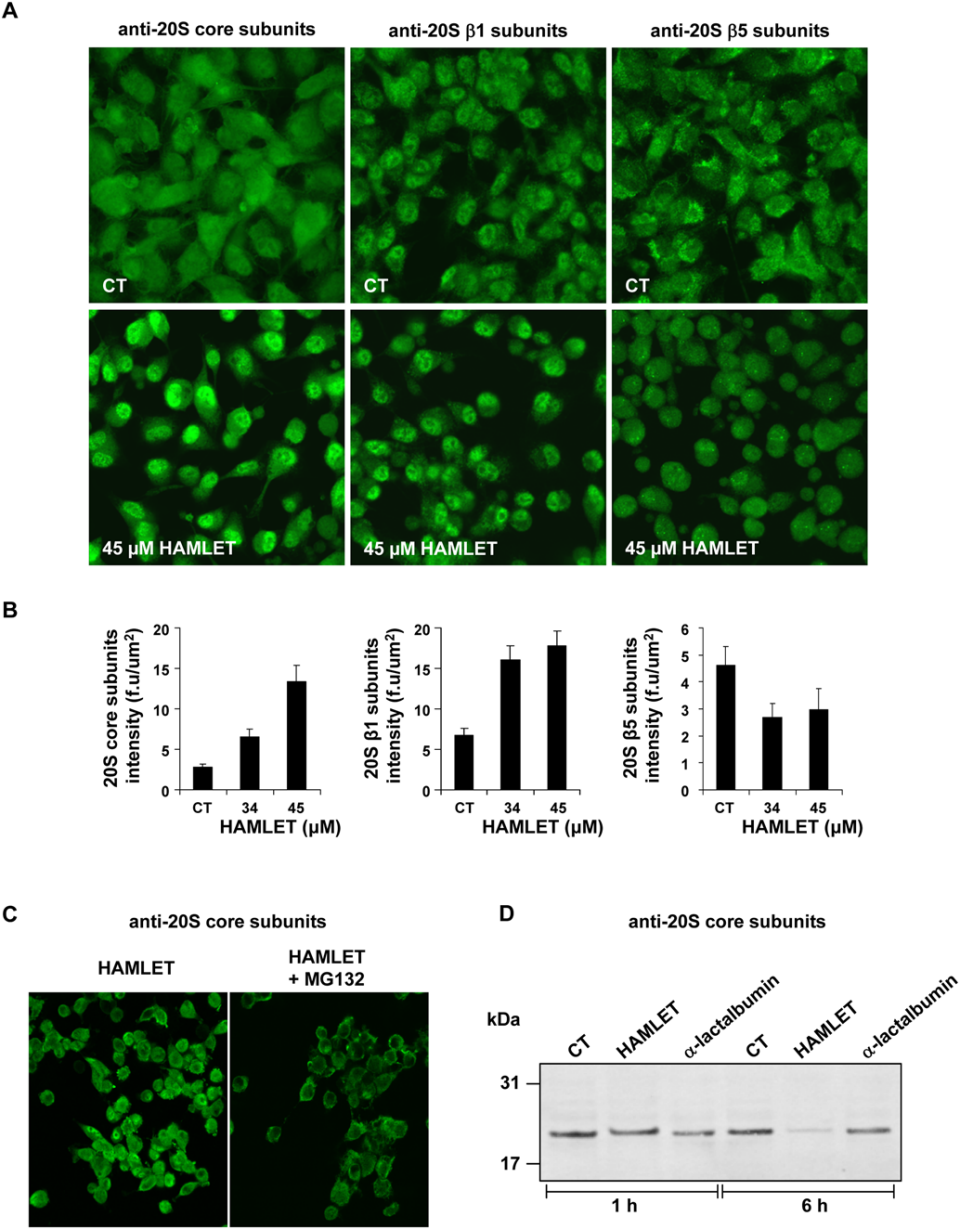
Changes in 20S proteasome staining after HAMLET treatment of carcinoma cells. (A–C) Changes in the staining of 20S proteasome subunits in A549 cells after HAMLET treatment (34 and 45 µM, 1 hour). Cells were stained with antibodies against the 20S proteasome core subunits (α5, α7, β1, β5, β5i and β7), antibodies against the β1 subunit or antibodies against the β5 subunit. (A) Confocal images showing increased core and β1 subunit staining, and decreased β5 subunit staining after HAMLET treatment (45 µM, 1 hour). (B) Fluorescence intensity was quantified in control and HAMLET-treated cells (34 and 45 µM, 1 hour). (C) The increase in core subunit staining after HAMLET was blocked by proteasome inhibition with MG132. (D) Western blot of cell extracts from cells treated with HAMLET (34 µM) for 1 and 6 hours, showing loss of 20S proteasomes staining after 6 hours. Antibodies against the 20S proteasome core subunits were used.

Confocal microscopy was used to visualize the change in 20S proteasomes structure, following HAMLET treatment. A increase in staining of 20S proteasome core subunits was observed after HAMLET treatment suggesting that the epitopes targeted by the polyclonal antibodies became more accessible (Figure 6A, B). This effect was inhibited by MG132 (Figure 6C). The effect was specific for HAMLET, as α-lactalbumin treatment did not alter proteasome staining (data not shown). A similar increase in staining after HAMLET treatment was observed using anti-β1 subunit antibodies (Figure 6A, B). In contrast, when using the polyclonal β5-subunit antibody, we observed a decrease in staining consistent with a loss of this subunit after HAMLET treatment (Figure 6A, B).

Alterations in proteasome structure were further examined by Western blots of total cell extracts from HAMLET-treated A549 cells (34 µM for 1 or 6 hours). In the control one main band of approximately 20 kDa was detected using antibodies against the 20S proteasome core subunits (Figure 6D). After 6 hours of HAMLET treatment, the intensity of this band was reduced.

The results of confocal microscopy and Western blot experiments suggest that the structure of 20S proteasomes is modified in response to HAMLET.

## Discussion

Partially unfolded proteins are usually degraded in the producing cell and the native, folded species is secreted or used for diverse intracellular functions. In some cases, however, folding is prevented by structural anomalies. HAMLET exemplifies a third alternative where a native, secreted protein readily unfolds in response to new environments and where this partially unfolded species then is internalized by target cells. In the case of HAMLET the partially unfolded protein is α-lactalbumin, which is prevented from reversion to the native state by binding to a fatty acid cofactor. We show here that HAMLET invades tumor cells and challenges the machinery that normally degrades partially unfolded proteins. The results suggest that HAMLET is recognized by the 20S proteasomes but resists degradation and forces the proteasomes to undergo structural modifications that inhibit their activity. We speculate that this process might contribute to the accumulation of HAMLET in tumor cells and to the cytotoxic response in tumor cells.

Unfolded proteins are a known threat to tissue homeostasis and yet, misfolding accompanies normal protein synthesis [3], [26], [27]. About 40% of newly synthesized peptides have errors, which prevent them from folding to the native state and many of these peptides are targeted to the proteasomes and successfully degraded [28]. Mutations may permanently hinder normal folding and in this case the risk of pathology increases as the unfolded proteins accumulate in the tissues causing aggregation and formation of insoluble fibrils and plaques [27]. One such protein is the amyloid beta-protein (A beta) and its precursor found in neurofibrillary tangle-containing neurons. A beta has been shown to selectively inhibit the chymotrypsin-like activity of the 20S proteasome leading to formation of inclusion bodies in neurodegenerative disorders such as Alzheimer's disease and Down's syndrome [5], [6]. The prion protein is thought to unfold extracellularly and the prions are cytotoxic, in part, by invading target cells [29], [30]. Disease-causing Prp^sc^ peptides were recently shown to inhibit the activity of 26S proteasomes, which require ubiquitin tags for degradation [7]. Pre-incubation with an antibody specific for aggregation-prone intermediates abrogated this inhibition.

HAMLET represents a different type of unfolded protein species but shares some features with the prions. The native protein is secreted by the mammary gland but can unfold in response to environmental change such as low pH in the stomach. Partially unfolded α-lactalbumin would normally revert to the native state under physiological conditions but the oleic acid-bound form in HAMLET remains stably unfolded and the complex invades tumor cells. This study shows that HAMLET binds directly to several subunits within the 20S proteasome. Such direct interactions of partially unfolded protein complexes and the proteasome have not previously been demonstrated. As a result, large amounts of partially unfolded α-lactalbumin may resist proteasome degradation, leading to HAMLET accumulation in tumor cells.

In addition, HAMLET triggered structural modifications of the 20S proteasomes. For example, HAMLET triggered the fragmentation of the α3 subunit, which is a key regulator of the proteasome gate. Previously, conformational changes of proteasome α subunits have been described but proteasome fragmentation in response to cellular agonists have not been reported. Co-crystallization studies of yeast 20S proteasomes with the 11S activator from *Trypanosoma brusei* suggested that the 11S activator opens the proteasome entrance port by modifications of the α subunits [31]. Functional studies in yeast confirmed this effect [32]. Furthermore, an N-terminal deletion of the α3 subunit has been shown to abolish proteasomal peptidase activity and to disorder the remaining pore residues [33]. The present study detected a structural modification of the α3 subunit, suggesting that direct effects of HAMLET on this subunit might cause further disordering of the proteasome. In addition, the structural α2, α6 and β3 subunits were modified, as were the catalytic β1 and β5 subunits. This gradual disintegration of the proteasome might include the 26S proteasome, especially since we also observed fragmentation of the 19S Rpt3 ATPase (data not shown). By confocal microscopy, alterations in subunit staining were observed after HAMLET treatment. There was a loss of β5 staining, compatible with the mass spectrometry data. In parallel, there was an increase in staining of β1 and other core subunits, suggesting structural modifications that make those subunits more available to the antibodies used.

We speculate that the inhibition of proteasome activity by HAMLET may be explained by the structural changes in the proteasome but the molecular basis of structural proteasome modification in response to HAMLET is not clear. Two scenarios may be proposed. Firstly, the proteasomes may self-destruct due to a dysfunctional interaction with the target. The protease resistance of the HAMLET complex might result in a prolonged enzymatic attack that eventually rebounds to include the proteasome itself. This hypothesis was supported by the mass spectrometry analysis since a chymotrypsin-cleaved fragment of proteasomal α2 subunit was observed after HAMLET treatment. Secondly, the proteasome structure might be modified by oleic acid as proteasomes are activated by low concentrations of surface-active molecules including detergents. Watanabe *et al.* have shown that low concentrations of oleic acid, linoleic and linolenic acids activate the chymotrypsin and peptidylgutamyl hydrolyzing activity of the proteasomes [34]. The lipid cofactor in HAMLET may thus participate in proteasome activation and possibly help to disturb proteasome structure.

So far, the failure to degrade unfolded proteins has mainly been described as a cause of cytotoxicity and disease. HAMLET has beneficial effects, however, due to its effects on tumor cells. The present study proposes that the entry of exogenous HAMLET exposes tumor cells to large amounts of partially unfolded α-lactalbumin and that the destructive proteasome response to HAMLET occurs mainly in tumor cells. While cellular response mechanisms might be shared between HAMLET and other unfolded protein species such as amyloid, there are major differences in the cellular spectrum. HAMLET appears to have struck a balance between beneficial and destructive mechanisms, which may be a key to tumor cell death.

## Materials and Methods

### Ethics statement

This study was conducted according to the principles expressed in the Declaration of Helsinki. The study was approved by the Medical Ethics Committee of the Lund University Medical Faculty, Lund, Sweden (decision number LU 456-96). Samples were obtained after written informed consent from the parents.

### Preparation of α−lactalbumin and HAMLET

α-Lactalbumin was purified from human milk and HAMLET was produced from α-lactalbumin as described [9]. Briefly, α-lactalbumin was partially unfolded by EDTA treatment and bound to oleic acid by ion exchange chromatography on a DEAE-Trisacryl M column (Pall BioSepra, Cergy-Saint-Christophe, France) preconditioned with oleic acid. When required, HAMLET or α-lactalbumin was conjugated to Alexa Fluor 568 according to the manufacturer's instructions (Molecular Probes Inc., Eugene, OR, USA).

### Cells

The human lung carcinoma cell line A549 (CCL 185, ATCC, Manassas, VA, USA), the human glioblastoma cell line D54 (gift from Darrel D-Bigner, Duke University, Durham, NC, USA) and the human kidney carcinoma cell line A498 (HTB44, ATCC) were cultured as described [8], [11], [35]. Human non-transformed astrocytes (CC 2565, Cambrex, La Jolla, CA, USA) were cultured according to the manufacturer's instructions. Human renal epithelial cells (HRTEC) were kindly provided by Dr. Diana Karpman (Lund University, Lund, Sweden). HRTEC were isolated from the kidney of a 3-year old boy as described [36] after written informed consent from the parents. IRB approval was obtained from the Medical Ethics Committee of the Lund University Medical Faculty, Lund, Sweden (decision number LU 456-96).

### Near-UV Circular dichroism spectroscopy

Lyophilized material was dissolved in 5 mM Tris, pH 8.5 to 70 µM. Near-UV CD spectra were collected at 25°C between 240 and 320 nm. The wavelength step was 1 nm, the response time was 8 sec and the scan rate 10 nm/min. An average of six scans is presented where the mean residue ellipticity, *θ*_m_ in deg×cm^2^×dmol^−1^, was calculated as previously described [37].

### Cell death assay

Trypan blue exclusion was used to measure viability. Cell suspension was mixed 3∶1 with 0.2% Trypan blue (Chroma Gesellschaft Schmid & Co, Stuttgart, Germany) in 0.9% NaCl and the number of living cells was counted in a Bürker chamber.

### Confocal fluorescence microscopy

Cells were detached from tissue culture flasks with Versene buffer (140 mM NaCl, 2.4 mM KCl, 8 mM Na_2_HPO_4_, 1.6 mM KH_2_PO_4_, 0.5 mM EDTA, pH 7.2) and grown on 8-well glass slides (Nalge Nunc, Naperville, IL, USA) overnight (50,000 cells/well). Cells were then washed twice with PBS, incubated at 37°C with 34 µM HAMLET (34 µM unlabelled HAMLET or 7 µM Alexa Fluor 568-labeled HAMLET and 27 µM unlabeled HAMLET) or 34 µM α-lactalbumin (34 µM unlabelled α-lactalbumin or 7 µM Alexa Fluor 568-labeled α-lactalbumin and 27 µM unlabeled α-lactalbumin) in serum-free medium. The slides were washed twice in PBS and fixed in 4% paraformaldehyde. For antibody stainings cells were then permeabilized in 0.1% saponin, 5% FCS, PBS for 20 minutes and incubated with polyclonal rabbit antibodies against the core subunits (α5, α7, β1, β5, β5i and β7) of the 20S proteasome (1∶100–500, Cat No. PW8155, BioMol, Denvon, UK), monoclonal mouse anti-β1 antibodies (1∶100, Cat No. PW8140, BioMol), polyclonal rabbit anti-β5 antibodies (1∶100, Cat No. PW8895, BioMol) and secondary goat anti-rabbit-Alexa Fluor 488 or goat anti-mouse-Alexa Fluor 488 antibodies (both 1∶500–1000, Molecular Probes Inc.). Cells were examined by confocal microscopy in an LSM 510 META system (Carl Zeiss, Jena, Germany). Confocal scans were recorded with identical settings in each experiment and optical sections were set to 1.0 µm. Quantification of fluorescence intensity and co-localization analysis was performed using the LSM META 510 software package (Carl Zeiss).

### Flow cytometry

10^5^ A549 cells in suspension were treated with 7 µM Alexa Fluor 568-labeled HAMLET and 27 µM unlabeled HAMLET or 7 µM Alexa Fluor 568-labeled α-lactalbumin and 27 µM unlabeled α-lactalbumin. After 1 hour of HAMLET treatment 5% FCS was added. The binding and uptake of HAMLET or α-lactalbumin was quantified by flow cytometry using a Becton Dickinson FACScalibur cytometer and CellQuest Pro Software (BD Biosciences, San Jose, CA, USA).

### In vitro binding assay of HAMLET and 20S proteasomes

20S proteasomes (6 µg, BioMol) were denatured and separated by SDS-PAGE (NuPAGE 12% Bis-Tris or 4–12% Bis-Tris gels, Invitrogen, Carlsbad, CA, USA) and stained with Amido black or silver staining. Separate gels were blotted to PVDF-Plus membranes (GE Osmonics, Minnetonka, MN, USA), which were blocked 15 minutes with Sat-1 buffer (6.1 g/l ethanolamine, 9 g/l glycine, 10 g/l polyvinylpirolidone, 25% methanol) and 15 min with Sat-2 buffer (6.1 g/l ethanolamine, 9 g/l glycine, 1.25 g/l Tween-20, 5 g/l gelatine hydrolysate, 25% methanol) and incubated with HAMLET (0.14 mM) over night at room temperature. The membranes were then blocked in 2% BSA and incubated with polyclonal rabbit anti-α-lactalbumin antibodies (1∶500, Sigma-Aldrich, St. Louis, MO, USA) and HRP-conjugated goat anti-rabbit antibodies (1∶500, DAKO Cytomation, Glostrup, Denmark). Bound antibodies were detected with ECL Plus Western Blotting Reagent (GE Healthcare, Little Chalfont, UK) and GelDoc equipment (Bio-Rad Laboratories, Hercules, CA, USA). Alternatively, membranes were incubated with ^125^I-labelled HAMLET which was detected by autoradiography.

### Protein array

The interaction between HAMLET and more than 8,000 human proteins was investigated with a ProtoArray® Human Protein Microarrays v4.0 using Invitrogen's Protein-Protein Interaction Profiling Service. Experiments were conducted at Invitrogen (Branford, CT, USA) as previously described [38]. Briefly, arrays were probed with Alexa Fluor 568-labelled HAMLET in two different concentrations (5 and 50 ng/µl) in duplicate. Interactions between HAMLET and the immobilized proteins on the arrays were evaluated by their Z-Score rank within the array and compared to the interactions observed on the negative control array. A total of eighty-seven proteins were identified as candidate interactors with HAMLET.

### Co-immunoprecipitation

A549 cells were washed, diluted to 10^6^/ml in serum-free medium and treated with HAMLET (36 µM) for 1 hour in 24-well culture plates (TPP, Trasadingen, Switzerland). After treatment cells were detached with Versene buffer (140 mM NaCl, 2.4 mM KCl, 8 mM Na_2_HPO_4_, 1.6 mM KH_2_PO_4_, 0.5 mM EDTA, pH 7.2), collected by centrifugation (400×g, 8 minutes) and lysed in ice-cold NP-40 buffer (20 mM Tris-HCl, pH 8.0, 100 mM NaCl, 1 mM EDTA, 0.5% NP-40) supplemented with Complete protease inhibitor cocktail (Roche Diagnostics, Mannheim, Germany). Lysates were incubated for 1 hour at 4°C with washed rec-Protein G-Sepharose 4B Conjugate beads (Invitrogen) to clear unspecifically binding proteins. After that α-lactalbumin antibodies (Cat No. A10-128A, Bethyl Laboratories, Montgomery, TX, USA) were added to half of the lysate (5 µg antibodies/ml lysate), the other half was used as control and both lysates were incubated with gentle agitation at 4°C. After 1 hour washed Protein G beads were added and the incubation was continued overnight. Beads with bound proteins were collected by centrifugation (12,000×g, 30 sec) and washed 3 times in lysis buffer. To release bound proteins beads were boiled for 5 minutes in LDS sample buffer (Invitrogen) with DTT (100 mM). Proteins were separated by SDS-PAGE and blotted onto PVDF-Plus membranes (GE Osmonics). Membranes were blocked with BSA and incubated with polyclonal rabbit antibodies against the core subunits (α5, α7, β1, β5, β5i and β7) of the 20S proteasome (1∶2,000, Cat No. PW8155, BioMol) and HRP-conjugated swine anti-rabbit antibody (1∶1,000, Cat No. P0217, DAKO Cytomation). Bound antibodies were detected with ECL Plus Western Blotting Reagent (GE Healthcare) and GelDoc equipment (Bio-Rad Laboratories).

### Proteolysis

Native α-lactalbumin, HAMLET or partially unfolded α-lactalbumin was subjected to proteolysis *in vitro* using enzymatic activities of the 20S proteasome. The protein samples were dissolved in 10 mM ammonium acetate, pH 7.5. Native α-lactalbumin was in the presence of 200 µM calcium acetate, whereas partially unfolded α-lactalbumin was in 200 µM EDTA. The mixtures were incubated with trypsin (E∶S 1∶50, by weight) and chymotrypsin (E∶S 1∶100, by weight) and degradation was detected by SDS-PAGE.

Degradation by 20S proteasomes was examined by incubating HAMLET or partially unfolded EDTA-treated α-lactalbumin (7 µM) with active 20S proteasomes (BioMol, 6 µg) for 2 hours. Samples were subsequently separated by SDS-PAGE and proteins visualized by silver staining. MG132 (Z-Leu-Leu-Leu-CHO, Cat No. PI102-0005, BioMol) was used to inhibit proteasomes activity.

### Enzyme assay of proteasome activity

Chymotrypsin activity was determined using the fluorogenic substrate Suc-Leu-Leu-Val-Tyr-7-amido-methylcoumarin (suc-LLVY-AMC, 50 µM to *in vitro* experiments and 25 mM to cell extracts, BioMol) which was added to 200 ng of 20S proteasomes (BioMol, in 20 mM Tris, pH 7.2, 1 mM EDTA, 1 mM DTT) or to 10 µg of cell extract (in 20 mM Tris-HCl, pH 7.5). The reactions were monitored at 37°C at 390/460 nm excitation/emission wavelengths using a Novostar spectrofluorometer (BMG, Offenburg, Germany). For *in vitro* experiments 50 µM of MG132 (Cat No. PI102-0005, BioMol) was used to inhibit proteasomes activity.

### Western Blot

#### In vitro mixing of HAMLET with 20S proteasomes

HAMLET or α-lactalbumin (7 µM) were mixed with 6 µg human erythrocyte 20S proteasomes (BioMol) in 20 µl reaction buffer (20 mM Tris, pH 7.2, 1 mM EDTA, 1 mM DTT) and incubated for 20 minutes at 37°C. The proteins were separated by SDS-PAGE (NuPAGE 12% Bis-Tris or 4–12% Bis-Tris gels, Invitrogen) and stained with Amido black. Separate gels were blotted to PVDF-Plus membranes (GE Osmonics). Membranes were blocked with 2% BSA and incubated with polyclonal rabbit antibodies against the core subunits (α5, α7, β1, β5, β5i and β7) of the 20S proteasome (1∶500, Cat No. PW8155, BioMol) and HRP-conjugated goat anti-rabbit antibodies (1∶500, DAKO Cytomation). Bound antibodies were detected with ECL Plus Western Blotting Reagent (GE Healthcare) and GelDoc equipment (Bio-Rad Laboratories).

#### 20S proteasome detection in cell extracts

Cell extracts were denatured, separated by SDS-PAGE (NuPAGE, 4–12% Bis-Tris gels, Invitrogen) and blotted onto PVDF-Plus membranes (GE Osmonics). Membranes were blocked with 5% non-fat dried milk and incubated with polyclonal rabbit antibodies against the core subunits (α5, α7, β1, β5, β5i and β7) of the 20S proteasome (Cat No. PW8155, BioMol) and HRP-conjugated swine anti-rabbit secondary antibody (Cat No. P0217, DAKO Cytomation). Bound antibodies were detected with ECL Plus Western Blotting Reagent (GE Healthcare) and GelDoc equipment (Bio-Rad Laboratories).

### Preparation of cell extracts

10^6^ A549 cells were treated with 34 µM HAMLET in 24-well culture plates (TPP). For proteasome inhibition, cells were pre-incubated for 1 hour with 5 µM of the reversible proteasome inhibitor MG132 (Cat No. PI102-0005, BioMol). After treatment, cells were detached with Versene buffer (140 mM NaCl, 2.4 mM KCl, 8 mM Na_2_HPO_4_, 1.6 mM KH_2_PO_4_, 0.5 mM EDTA, pH 7.2), collected by centrifugation (400×g, 8 minutes) and lysed in ice-cold NP-40 buffer (20 mM Tris-HCl, pH 8.0, 100 mM NaCl, 1 mM EDTA, 0.5% NP-40) supplemented with Complete protease inhibitor cocktail (Roche Diagnostics). The DC protein assay (BioRad) was used to quantify total protein concentration.

### Matrix-assisted laser desorption ionization, time-of-flight (MALDI-TOF) mass spectrometry

After *in vitro* mixing of HAMLET and human erythrocyte 20S proteasomes (BioMol) mixtures were separated by SDS-PAGE (see details above). Bands of interest were excised, trypsinized and further analyzed by mass spectrometry using a M@LDITM-LR HT instrument (Micromass, Manchester, UK) and the MASCOT search engine. MALDI-TOF mass spectrometry was also performed on the HAMLET and proteasome mixtures without previous SDS-PAGE or trypsinization. For protein analysis mass spectra were calibrated externally and for peptide analysis internal calibration from trypsin autolysis peaks was used. Peptides were further analyzed with FindPept (http://www.expasy.ch/tools/findpept.html).

### Statistical analysis

Groups were compared with Student's t-test.
